# Novel Claw‐shaped Bone Plate in Complex Unstable Scapular Neck and Body Fractures: Comparison with Reconstruction Locking Plate

**DOI:** 10.1111/os.13766

**Published:** 2023-05-24

**Authors:** Huiming Shi, Kun Zhang, Yuanjun Hu, Wei Wu, Ning Liu, Haixia Lu

**Affiliations:** ^1^ School of Basic Medical Sciences Xi'an Jiaotong University Xi'an China; ^2^ Traumatic Orthopaedics Department Hanzhong Central Hospital Hanzhong China; ^3^ Orthopaedics Department of Xi'an Honghui Hospital Xi'an China

**Keywords:** Claw‐shaped bone plate, Intermuscular approach operation, Osteosynthesis, Plate, Scapula fracture

## Abstract

**Objective:**

For complex and unstable scapular fractures requiring simultaneous fixation of the glenoid neck, the lateral margin of the body, and/or the scapular diaphysis, reconstruction locking plate is difficult to achieve satisfactory fixation. In order to optimize the fixation effect, the newly designed claw‐shaped bone plate was designed for fixing such fractures. We also evaluate the clinical effects and follow‐up at an average of 1 year after treatment in scapular internal fixation by using reconstruction locking plate and claw‐shaped bone plate in complex unstable scapular body and glenoid neck fracture.

**Methods:**

A retrospective study was conducted from 2018 to 2021, thirty‐three patients (27 males and six females) who were defined unstable scapular fractures by Ada–Miller. Fifteen patients (52.86 ± 8.26 years) received claw‐shaped bone plate and 18 cases (51.61 ± 11.31 years) received reconstruction locking plate with the intermuscular approach. The clinical effect was evaluated based on the operation time, intraoperative blood loss, surgical complications, clinical healing time and Constant–Murley score (CMS). The data analysis by Student *t*, Mann–Whitney *U* test and Pearson's chi squared test.

**Results:**

Compared with reconstruction locking plate, the claw‐shaped bone plate showed shorter operation time (102.73 ± 18.43 min vs. 156 ± 37.53, *P* < 0.0001), higher CMS (94.00 ± 4.07 vs. 89.88 ± 5.42, *P* = 0.02) and no differences between the two groups regarding intraoperative blood loss (208.00 ± 96.45 mL vs. 269.44 ± 120.21, *P* = 0.12) and clinical healing times (9.96 ± 1.52 vs. 10.05 ± 1.67, *P* = 0.87). Follow‐up were conducted at first, third, 6 and 12 months after surgery. The operation was successful in all patients with no intraoperative complications.

**Conclusions:**

For the treatment of complex and unstable scapular neck body fractures, the application of claw‐shaped bone plate demonstrated short operation time, better stability of the fracture block, and higher CMS. In the intraoperative and postoperative follow‐up showed better clinical results and rehabilitation effects.

## Graphical abstract

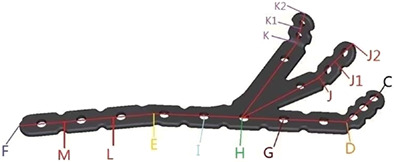

## Introduction

In 1799, Karl August Vogt was the first study scapula fractures.^1^, ^2^ Scapula fractures primarily caused by high‐energy direct impacts and usually accompanied by major additional injuries.^3^ According to the US National Trauma Data Bank, the rate of scapular fractures increased substantially from 1% to 2.2% in a single decade from 2002 to 2012.^4^ Scapula fractures account for 3%–5% of shoulder fractures, 0.5%–1% of systemic fractures, and 62%–98% of scapula body and neck fractures.^5^, ^6^, ^7^, ^8^ Due to the unique mechanical and biological environment of the scapula, the vast majority of scapula fractures show minimal displacement and will heal without dysfunction.^9^, ^10^, ^11^ In a long‐term follow‐up study of non‐operatively treated extra‐articular scapular fractures, half of the patients exhibited shoulder symptoms.^12^ Tatro *et al*.^13^ compared the surgical and non‐surgical treatment of scapular fractures and found excellent outcomes in the surgical group. Nowadays, surgical treatment is recommended as the first choice with comminuted displaced fractures, glenoid neck and fossa fractures.^14^ Plates are the most common implant in the surgical treatment of scapular fractures. Various osteosynthesis materials, such as reconstruction locking plate and screws can be used in the operative treatment of scapula fractures.^15^, ^16^ Due to the thinness of the scapula at the infraspinatus fossa the screws used for the plates must be short, which may result in plate failure.^17^ The osteosynthesis material also should provide stable fixation and not complicate the reattachment of muscles.

In complex unstable scapular fractures, it is difficult to stabilize the fracture after reduction, and pseudo‐joints or deformities form easily during conservative treatment. So surgery is usually required for complex unstable scapular fractures. Posterior approach surgery is commonly performed in complex unstable scapular body and neck fractures,^1^, ^18^, ^19^ and internal fixation is generally achieved with various osteosynthesis plates and screws.^16^, ^19^, ^20^, ^21^ Integrated fixation of the glenoid, neck, body, and scapula to obtain stable fixation is paramount to postoperative recovery.^20^ In the surgical procedure, limited by the space of the glenoid and neck, the reconstruction plates can only fix the main fracture mass at the lateral margin of the neck. Multiple screws fix the glenoid and neck with limited stability. The lack of fixation can only be offset by restricting movement and delaying exercise time, but it will affect postoperative functional recovery. Based on the complex anatomical structure of the scapula, the reconstruction plate is difficult to meet the fixation requirements for complex unstable scapular fractures, and there is a lack of other convenient and stable plates. Therefore, in this study, we designed a new claw‐shaped plate for complex unstable scapular fractures.

The objective of this retrospective cohort study was to: (i) propose this new design of bone plate which will help to integrate the scapula and makes up for the shortcomings of the reconstruction locking plate, and will be of great significance for clinicians; and (ii) evaluate the clinical effects between reconstruction locking plate and claw‐shaped bone plate.

## Materials and Methods

### Study Design

Between 2018 and 2021, retrospective review 33 patients with severe displaced scapular body and neck fractures were treated by claw‐shaped bone plate and reconstruction locking plate in the Traumatic Orthopedics Department of Hanzhong Central Hospital. Informed consent was obtained from all individual participants and all data were analyzed anonymously. This study was ethically audited and met the requirements of the Helsinki Declaration, as revised in 2013, and approved by the ethics committee of the Hanzhong central Hospital (No. IRB2018‐S).

### Patients

During the study periods, we collected 33 patients who had presented at the hospital with complex unstable scapular neck and body fractures. Based on the Ada–Miller classification, comminuted scapular fractures involving two or more classifications were prone to displacement or re‐displacement after reduction. These combinations of fracture types are defined as complex unstable fractures. There were four mixed type IB and IV fractures, one mixed type II and III fracture, 15 mixed type II and IV fractures, seven mixed type mixed type I, II, and IV fractures, two mixed type II, III, and IV fractures, and four mixed type I, II, III, and IV fractures.

The inclusion criteria were type IB, II, III, IV according to the Ada–Miller classification and containing the distal section of the shoulder blade. The exclusion criteria were pathological, deformed scapula fracture. Finally, 15 patients in the claw‐shaped bone plate group and 18 in the reconstruction locking plate group were eligible for this study, and they were followed‐up for at least 12 months, no patient dropped out during this period.

The indications for surgery were as follows^7^, ^22^, ^23^, ^24^: (i) collapse or displacement of joint surface ≥4 mm; (ii) lateral margin fracture displacement ≥20 mm; (iii) angulation ≥45°; (iv) glenopolar angle ≤22°; and (v) lateral margin displacement ≥15 mm with angulation ≥30°. Surgery was indicated if at least one of the above criteria was met.

### Design of Bone Plate

The claw‐shaped bone plate was designed with three proximal claws: the first claw fixes the glenoid neck, while the second and third claws fix the distal mesoscapula (Patent ID: ZL201820117876.8). The distal part of the plate fixes the outer area of the scapula while simultaneously forming a triangular mechanical support. There are three claws in the basic design, while the claws can be tailored into two claws and one claw according to the fracture situation of the specific case. At the same time, the length of any claw can be tailored according to the needs of fracture fixation during specific surgery.

Computed tomography (SOMATOM Definition Flash, Siemens, Munich, Germany, scan thickness 1.0 mm, 128 rows) scans with three‐dimensional reconstructions (3D‐CT scans) were ordered before surgery. Based on the data, fracture model and healthy side mirror model were constructed using a three‐dimensional (3D) printer software studio (Xijing Zhengwo Medical 3D Printing Cloud Terminal, reconstruction software: Mimics17.0). The fracture reduction and fixation strategy were designed according the 3D fracture and mirror model. The claw‐shaped bone plate or reconstruction locking plate was used according to the 3D mirror model. Polylactic acid (WeinanDing xinChuang xinZhizao Technology Co Ltd, Beijing, China) was used in the 3D printing model.

**Fig. 1:**
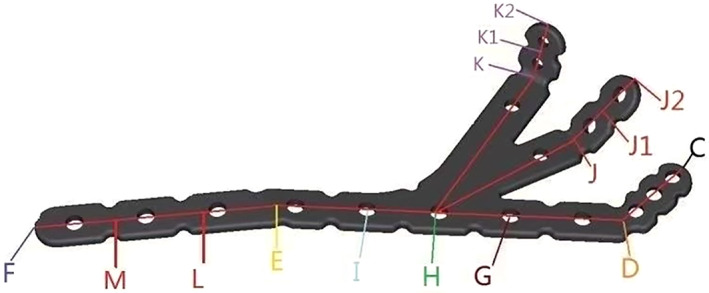
The design drawing of claw‐shaped bone plate. The CD segment is the fixed glenoid segment, the DE segment fixes the lateral edge of the neck, the EF segment fixes the inferior scapular angle extending from the lateral margin, and the H–J–J2 segment and the H–K–K2 segment fix the distal scapular segment. The distance between G/H/I points and D points is 3, 4, and 5 cm. M/L are the third equinox of EF, and J1/K1 are the midpoints of JJ2/KK2, respectively.

**Fig. 2:**
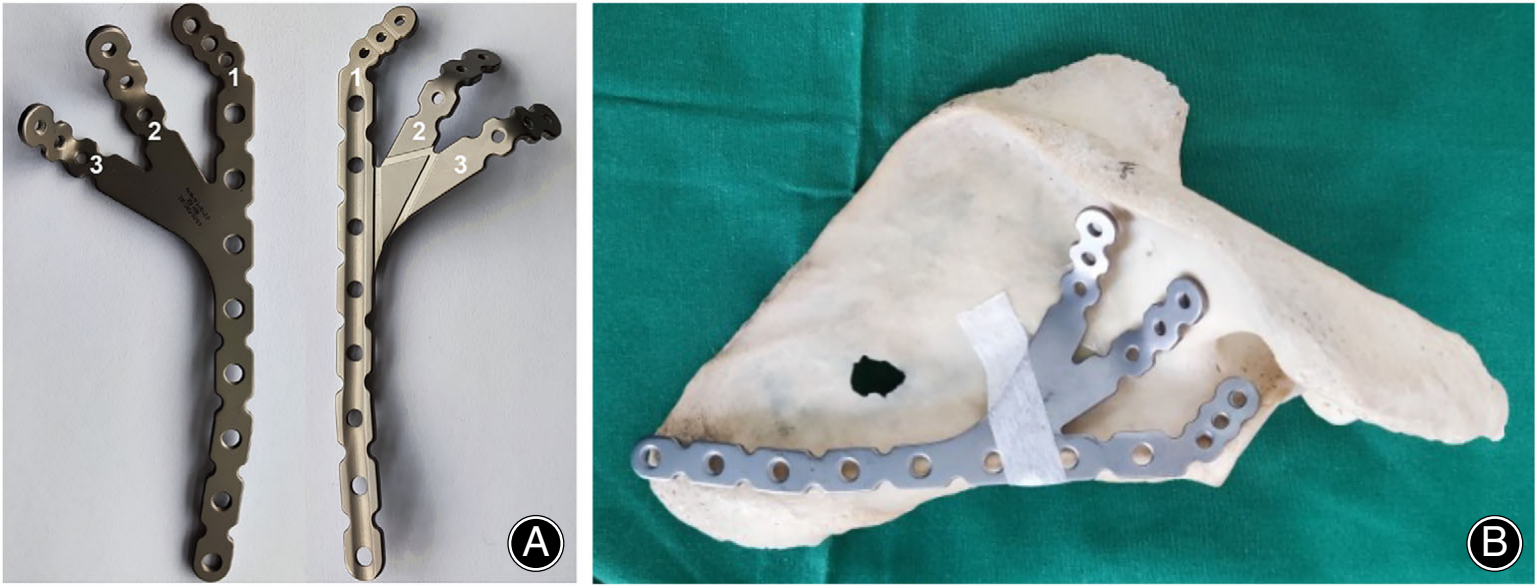
(A) The claw‐shaped bone plate (front side and reverse side). Among them, 1, 2, and 3 identify the first, second, and third claws of the claw plate, respectively. (B) The claw‐shaped bone plate adheres to the surface of the scapular.

The claw‐shaped bone plate (Figs 1 and 2A,B) design parameter: thickness of 2.0 mm, width of claw is 10 mm, the pitch of hole are 16, 6, 10 mm respectively, the angle of claw ∠CDE (128.61° ± 6.30°), ∠DEF (173.01° ± 4.45°), ∠HJJ2(133.43° ± 7.47°), ∠HKK2(123.55° ± 7.84°). The diameter of the screw hole: outer edge (3.5 mm), claw (2.7 mm); the hole spacing: outer edge (16 mm) and the first, second, and third claws are 6, 10, 10 mm respectively. The claw‐shaped bone plate from Beijing Best Biotech Co., Ltd. (Beijing, China): model number AZX‐LL, project code 9797 was used. The reconstruction locking plate was from the Beijing Best Biotechnology Co., Ltd. (Beijing, China): model AZX‐LL, thickness of 3.0, and 3.5 mm locking nails.

### Surgical Technique

#### Anesthesia and Position

All patients were placed lateral decubitus position on healthy side and performed under general anesthesia.

#### Surgical Approach

Using the modified Judet approach,^25^, ^26^ the incision started from the acromion, proceeded inward along the scapula, and then curved toward the inferior angle of the scapula. The shoulder joint was adducted to keep the axillary nerve and radial nerve away from the operation area, separating the fascial space to ligate the artery to expose the parts of the scapular body and glenoid neck fracture. Establishing a channel from the lateral edge of the scapula to the scapular‐neck and the lateral border of the scapula‐mesoscapula channel to implant the plate and fix the fracture.

#### Placement of the Claw‐shaped Bone Plate

During the operation, the major fractures of the lateral border and glenoid neck were reduced and were temporarily fixed by Kirschner wire. Based on the preoperative plan, the claw‐shaped bone plate was implanted from the teres minor of the lateral edge of the scapula and the gap of the infraspinatus. The first claw was implanted in the neck of scapula through the channel of lateral edge of the scapula to the scapular‐neck, while the second and third claws were implanted through the lateral edge of scapula‐mesoscapula channel, and the claw‐shaped bone plate was attached to the distal edge of scapula. Non‐major fractures were not fixed during surgery.

#### Placement of the Reconstruction Locking Plate

The surgical approach of the reconstruction locking plate group was basically the same as the claw‐shaped bone plate group. After the exposed fracture was reduced, fixed by the clamp or Kirschner wires temporarily, and the shaped reconstruction locking plate was selected and used, according to the status of fracture. Reconstruction locking plates were mainly used to fix the lateral edge of the neck and body, and combined with scapular ganglia fractures which needed to be fixed with another plate. Combined with glenoid neck fracture, if the fracture end is less than 2.0 cm away from the inferior glenoid margin, it is difficult for the reconstruction locking plate to attach to the posterior glenoid margin and the lateral neck margin, which can only be fixed with screws, and it is necessary to attach Kirschner wires or screw to strengthen the glenoid neck fixation. For comminuted glenoid neck fractures, poor fixation stability requires additional postoperative external fixation immobilization protection.

After the plate was implanted, drainage was placed and the incision was closed in a layered fashion. All patients included in the study signed informed consent forms, and the surgery was performed by Huiming Shi.

#### Postoperative Rehabilitation and Follow‐up

Postoperative elbow suspension was used for 2 weeks to protect the affected limb. After the first week, gradual shoulder joint function exercises and passive movement (shoulder joint pendulum exercise) were instructed. After 2 weeks, the patients completed shoulder shrugging and passive abduction activities and increased the range of joint activity. Shoulder joint lifting activity was started after 3 weeks. Active shoulder joint exercise was gradually introduced after 4 weeks. Shoulder muscle strength and endurance training was started after 8 weeks. Outpatient follow‐up was conducted at first, third, 6 and 12 months after operation respectively and the X‐ray in the first, 12 months.

### Outcomes

There are several observational measures in this study, such as the operation time, intraoperative bleeding volume, surgical complications, fracture healing time, the Constant–Murley score (CMS) and so on. The primary outcomes of this study were the operation time and intraoperative bleeding volume. Operation time is calculated from the beginning of incision to the end of the operation. Intraoperative bleeding volume measured by the negative pressure wound therapy devices and the weighing medical gauze (removed intraoperative infusion and normal saline). The same nurse was used during surgery to ensure the accuracy and reliability of relevant data collection. The secondary outcomes were surgical complications. Complications could include shock, hemorrhage, wound infection, deep vein thrombosis and pulmonary embolism, lung (pulmonary) complications, urinary retention, and reaction to anesthesia. Fracture healing time is the time from the time of injury to the time when all of the radiographic criteria and clinical criteria were met.^27^ CMS was assessed at the last follow‐up. The CMS is a 100‐points scale composed of a number of individual parameters. These parameters define the level of pain and the ability to carry out the normal daily activities of the patient.^28^

### Statistical Analysis

The data were analyzed by SPSS 26.0 statistical software. The continuous data were expressed as mean ± standard deviation (*x* ± *s*), while the counting data were expressed as number (*n*) and rate (%). Normally distributed data were analyzed by using the Student *t* test. Non‐normally distributed data are presented as median (range) and were analyzed by using the Mann–Whitney *U* test. Pearson's chi squared test were used to analysis categorical data (Table 1).

**TABLE 1:** The baseline characteristics of the patients (*n* = 33)

Characteristics	Claw‐shaped bone plate (*n* = 15)	Reconstruction locking plate (*n* = 18)	*t*	*χ* ^2^	*P*
Age (years), mean ± SD	52.86 ± 8.26	51.61 ± 11.31	0.35	‐	0.72
Gender, *n* (%)	‐	‐	‐	0.06	0.80
Male	12	15	‐	‐	‐
Female	3	3	‐	‐	‐
Location of injury, *n* (%)	‐	‐	‐	0.55	0.46
Left	9 (60)	13 (72)	‐	‐	‐
Right	6 (40)	5 (23)	‐	‐	‐
Scapula classification (Ada‐Miller)	‐	‐	‐	7.5	0.48
IB + IV	‐	4	‐	‐	‐
II + III	1	‐	‐	‐	‐
II + IV	8	7	‐	‐	‐
IA + II + IV	1	2	‐	‐	‐
IB + II + IV	1	3	‐	‐	‐
II + III + IV	1	1	‐	‐	
IC + II + III + IV	2	1	‐	‐	‐
IB + II + III + IV	1	‐	‐	‐	‐
Cause of injury, *n* (%)	‐	‐	‐	4.21	0.23
Traffic accident injuries	5 (34)	11 (61)	‐	‐	‐
Fall injuries	6 (40)	2 (11)	‐	‐	‐
High fall	2 (13)	2 (11)	‐	‐	‐
Bruises	2 (13)	3 (17)	‐	‐	‐

*Notes*: The values are the means ± SD, *n* = 33. the age between the two group were tested by student *t* test (*P* = 0.72). the gender (*P* = 0.80), location of injury (*P* = 0.46), scapula classification (Ada–Miller) (*P* = 0.48) and cause of injury (*P* = 0.23) between the two groups were tested by Pearson's chi squared test.

## Results

### General Results

Thirty‐three patients were treated by the surgery without intraoperative complications. The 33 patients included 27 males and six females. The scapula fractures primarily occurred in middle‐aged and elderly men and mean age were 52.18 ± 9.91 (range, 33–70) years and the left or right side were 22 and 11 respectively. The left scapula is prone to complex unstable fracture in middle‐aged and elderly people after injury and the types of Ada–Miller were mainly mixed type II, III and IV. In the group of claw‐shaped bone plate, there were 12 men and three women and the age (mean ± SD) was 52.86 ± 8.26 years old, location of injury left /right: 9/6. In the reconstruction locking plate group, there were 15 men and 3three women and the age (mean ± SD) was 51.61 ± 11.31 years old, location of injury left /right: 13/5. There were no significant differences in age, gender, location of injury, scapula classification (Ada–Miller) and cause of injury between two groups. The baseline characteristics of the patients are listed in Table 1.

### Intraoperative Results

During the operations, The surgical duration (mean ± SD, min) was 102.73 ± 18.43 and 156.22 ± 37.53 respectively, and the blood loss (mean ± SD, mL) was 208 ± 96.45 and 269.44 ± 120.2. Based on the data analysis from intraoperative, there were significant differences in operation time (*P* < 0.0001) and no significant intraoperative blood loss difference (*P* = 0.12) between the two groups (Table 2).

**TABLE 2:** Comparison of blood loss, operation time between two groups

Indexes	Claw‐shaped bone plate (*n* = 15)	Reconstruction locking plate (*n* = 18)	*t*	*P*
Intraoperative blood loss (mL), mean ± SD	208 ± 96.45	269.44 ± 120.21	1.59	0.12
Operation time (min), mean ± SD	102.73 ± 18.43	156.22 ± 37.53	5.02	<0.0001

*Notes*: The values are the means ± SD, *n* = 33. The blood loss (mL) and the operation time (min) in two groups were tested by student *t* test. *P* < 0.0001, Operation time of claw‐shaped bone plate versus Operation time of reconstruction locking plate.

### Fracture Healing and Follow‐up

No case was lost to follow‐up. All patients had achieved primary incision healing and follow‐up for at least 12 months. The CMS assessed shoulder joint function, and the claw‐shaped bone plate group showed higher CMS (94.00 ± 4.07 vs. 89.88 ± 5.42, *P* = 0.02), compared with the reconstruction locking plate group. The healing time (mean ± SD, weeks) was 9.96 ± 1.52 and 10.05 ± 1.67 respectively, there were no significant difference between the two groups (Table 3).

**TABLE 3:** Comparison of Constant–Murley score (CMS) (12 months) and healing time between two groups

Indexes	Claw‐shaped bone plate (*n* = 15)	Reconstruction locking plate (*n* = 18)	*t*	*P*
Constant–Murley score	94 ± 4.07	89.88 ± 5.42	2.42	0.02
Healing time (weeks)	9.96 ± 1.52	10.05 ± 1.67	0.15	0.87

*Notes*: The values are the means ± SD, *n* = 33. The CMS and healing time (weeks) in two groups were tested by student *t* test. *p* < 0.05, CMS of claw‐shaped bone plate versus CMS of reconstruction locking plate.

### Application of the Claws in the Claw‐shaped Bone Plate Group

**Fig. 3:**
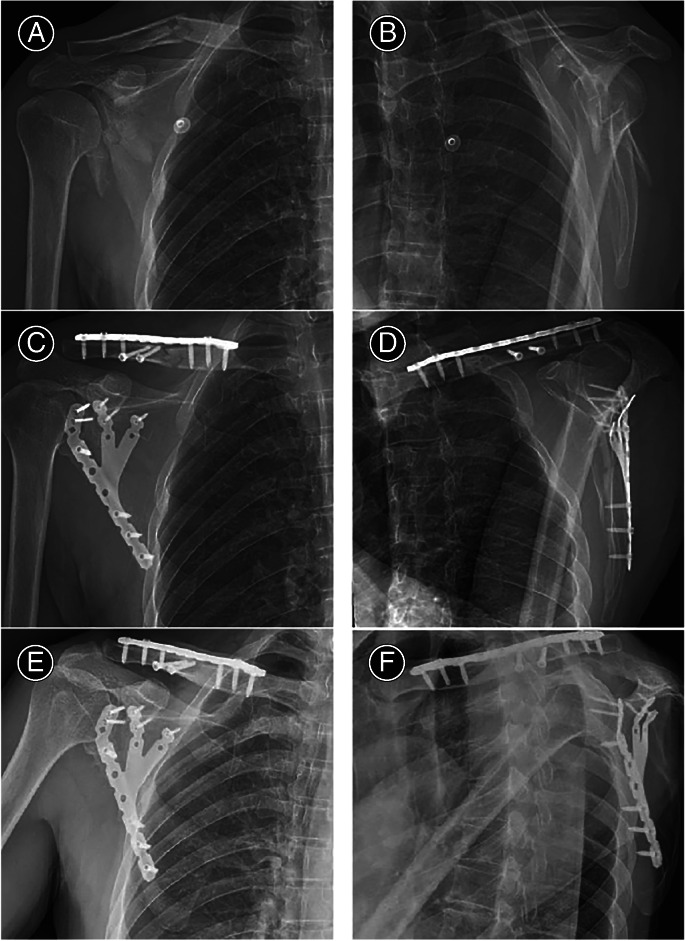
Images of X‐ray from a 48‐years old male patient with scapular fractures (II + III + IV), the claw‐shaped bone plate (anterior view and posterior view). (A, B) Preoperative schematic diagram of X‐ray. (C, D) X‐ray of the first month after postoperative. (E, F) X‐ray of the 12 months after postoperative (unless specified, the results of this study were from this typical case).

**Fig. 4:**
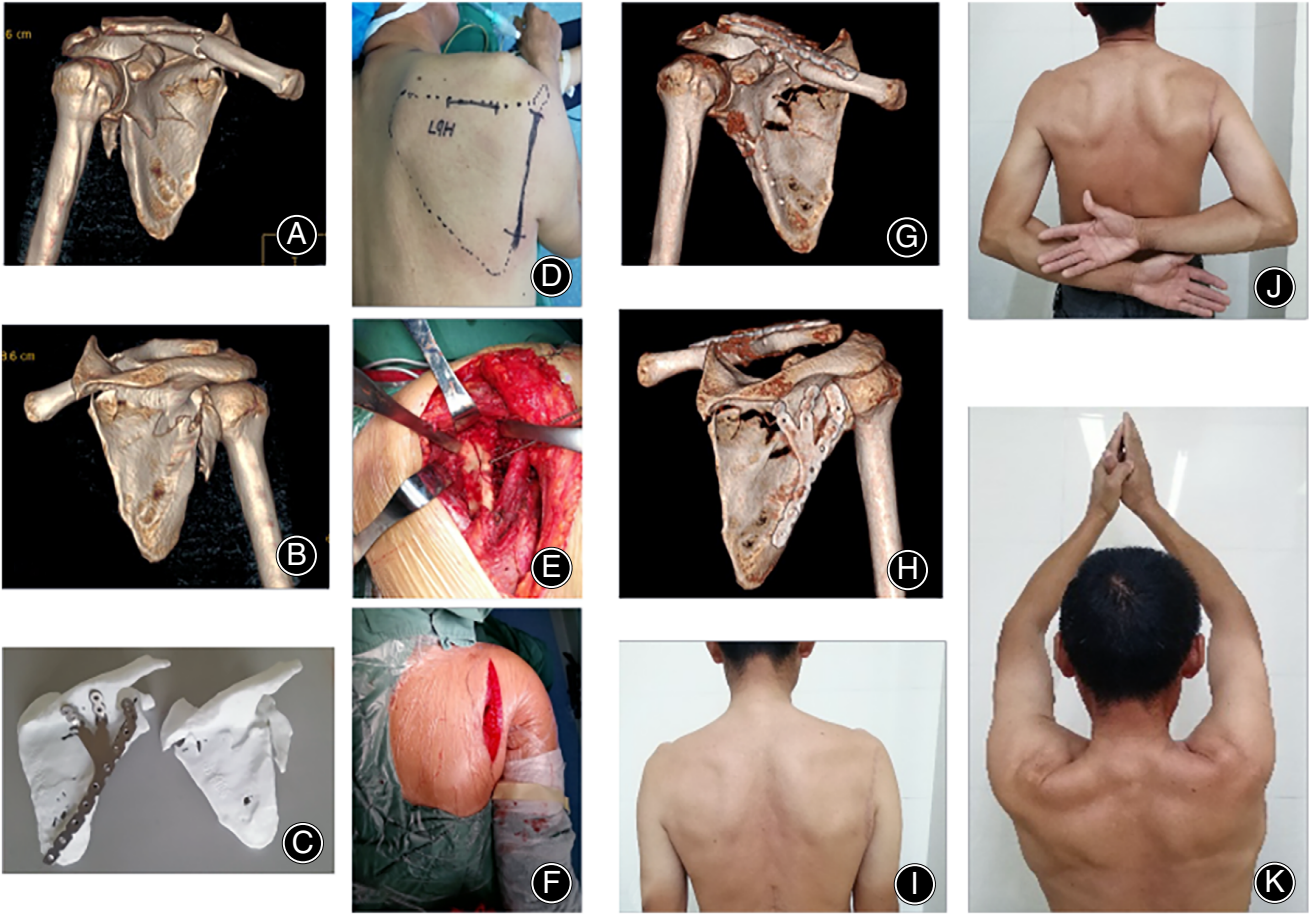
Images from a 48‐year old male patient with scapular fractures (Ada–Miller type: II + III + IV) and the recovery result. (A, B**)** Preoperative 3D CT images (anterior view and posterior view) showing the scapular fracture. (C) 3D printing fracture model and mirror model. (D**)** Incision using the lateral edge approach. (E) Reduction and fixation of intermuscular approach. (F**)** The incision of surgical. Patient recovery results. **(**G, H) Postoperative 3D CT images (anterior view and anterior view). (I–K) Physical examination and general appearance at 6 months after operation shown that the function had recovered well.

In claw‐shaped bone plate group surgical treatment, seven patients were immobilized using plate with three claws, seven cases were treated with plate of two claws, only one patient used the one claw plate (Table 4). The typical case of the claw‐shaped bone plate group is shown in Figs 3 and 4.

**TABLE 4:** Application the claws in this study (*n* = 15)

	Claw‐shaped bone plate (*n* = 15)
Number of claws^a^, *n* (%)	‐
Three claws	7 (46.7)
Two claws	7 (46.7)
One claw	1 (6.6)

^a^ The number of claws is the number of bony plates used to fix the glenoid and neck, trimmed according to the specific condition of the fracture.

### Complications

No neurovascular injury, peri‐implant fracture, deep infections, screw prolapse and plate rupture, or other severe complications were observed in either group.

## Discussion

We retrospectively analyzed the clinical effects of claw‐shaped bone plate and reconstruction locking plate in the treatment of complex unstable scapular fractures with internal fixation. The claw‐shaped bone plate fixation the major fracture blocks through the intermuscular approach required less operation time and achieved better shoulder function (which was assessed by CMS) compared with the reconstruction locking plate. There was no significant time difference in rehabilitation, however it seemed better in the claw‐shaped bone plate group. No intraoperative and postoperative complications were observed in this study.

### Assessment of Various Bone Plates in Scapular Fracture

The common internal fixations include 3.5‐mm reconstruction locking plate, and microplate, 2.7 mm reconstruction locking plate were auxiliary in the surgery progress.^29^ The other infrequently used plate such as calcaneous deformed plate,^30^ hook plate.^31^ In this research, the main objective evaluated the clinical effect of new claw‐shaped bone plate and compared the functional outcomes between claw‐shaped bone plates and the reconstruction locking plates in the complex unstable scapula fractures.

The reconstruction locking plate is mainly based on the fixation of the main fracture fragments, subjecting to the limitation of scapula glenoid and neck.^32^ Generally, the 3.5 mm aperture reconstruction of the bone plate is mainly based on fixing the main fracture piece of the lateral edge of the neck of the simple body, which is limited by the space limitation of the glenoid neck and the shaping of the bone plate itself. Glenoid neck fractures (defined by a distance between the inferior glenoid and fracture line of less than 2 cm), only one screw is allowed which is difficult to fix, resulting in limited stability.^19^ Although additional constrained motion after surgery can balance the deficiency in immobilization by delaying exercise time, postoperative functional recovery is still affected. For comminuted fractures, the addition of medial margin and/or scapular plate fixation can increase the fixation strength.^16^ Most of the scapula is thin, and the effective fixation area of the additional bone plate is limited. Thus, implantation is difficult, the increase in fixation strength is limited, and large tissue dissection is required. Although the Y‐shaped plate allow the integrated fixation of the lateral margin of the scapula, glenoid neck area, and mesoscapula, these plates are still inadequate in matching with scapula.^32^ Other plate designs such as the glenoid neck plate, glenoid‐lateral margin plate, scapular‐medial margin plate, subscapular angle plate, and integrated frame plate have different effects because they are designed for use in different fracture types and combined with different surgical approaches.^33^, ^34^, ^35^ The claw‐shaped bone plate integrates the lateral margin of the neck the glenoid and the distal segment of the scapular spine, solved the confusion of selecting the appropriate bone plate for the complex unstable scapular fracture. As illustrated in the typical case, the claw‐shaped bone plate is consistent with the preoperative 3D model. Postoperative imaging (Figs 3 and 4) showed that all major fractures were fixed well and triangular supports were stable. The operative time, intraoperative blood loss and postoperative function indicate that the claw‐plate bone plate has the characteristics of convenient, stable fixation, and satisfactory postoperative function. It is an optimal choice for complex and unstable scapular fractures.

### Clinical Surgical Treatment of Scapular Fracture

In recent years, arthroscopic treatment of scapular fractures as a new treatment has achieved satisfactory results.^36^ These include type IA scapular glenoid fractures.^37^ However, for comminuted fractures of the lateral margin of the scapula, including pelvic neck fractures, midline fractures of the scapula complex and unstable scapular fractures. Open reduction and internal fixation remain the preferred treatment modalities. According to the Ada–Miller classification, the fractures in this study were classified as mixed type I + II + III + IV fractures.^38^, ^39^ In this type of fracture, common fixation methods (fixation of the lateral margin, including the glenoid) are difficult to obtain stable fixation.^17^, ^40^ Therefore, fixing the mesoscapula was necessary to form a triangle and achieve stable fixation. The lateral margin of the neck, body of the scapula, glenoid, and mesoscapula are the main biomechanical regions of the scapula;^41^ only by fixing the main fracture blocks in these areas can the scapula fracture be stabilized and fixed.^42^ In the present study, the short‐term (3 months) shoulder joint function after claw‐shaped bone plate internal fixation surgery was better than with the straight reconstruction locking plate. The intermuscular approach combined with the claw‐plate implantation can reduce tissue damage and bleeding and shorten the operation time, which is helpful to reduce postoperative pain.

### Strengths and Limitations

For complex unstable fractures, combined with glenoid and neck and/or distal segment of scapular spine, the new design of claw‐shaped bone plate provides an adaptive and optimized option for fixing the main fracture block though the intermuscular approach and lays a foundation for obtaining good postoperative shoulder joint function. Some limitations of this study are that the claw‐plated bone plate of the scapula is still not suitable for partial fractures, such as simple lateral margin or scapular ganglia fractures, and simple glenoid neck fractures. The number of cases was small and the representativeness of the studies was limited. Thus, further studies will larger sample sizes or multi‐center joint studies are needed to demonstrate the reproducibility of the conclusions.

### Conclusion

For complex and unstable fractures of the scapula body and neck, the claw‐shaped bone plate was selected to fix the main fracture block through the intermuscular approach exposure, which is practical and convenient, provides stable fixation, reduces trauma, and produces a satisfactory clinical treatment effect.

## Author Contributions

Huiming Shi proposed the concept, analyzed the data and wrote the article; Kun Zhang guided the operation; Haixia Lu guided the research framework and helped the article writing; Yuanjun Hu, Wei Wu and Ning Liu participated in the data collection and collation.

## Ethics Statement

This research was approved by the ethics committee of Hanzhong central Hospital (IRB2018‐S). All participants agreed with the data and publication of the manuscript. Informed consent was obtained from all subjects. This study was ethically audited and met the requirements of the Helsinki Declaration, as revised in 2013.

## Consent for Publication

Written informed consent was obtained from all participants.

## Data Availability

The datasets used and/or analyzed during the current study are available from the corresponding author on reasonable request.
